# Echocardiographic Measures of Cardiac Structure and Function Are Associated with Risk of Atrial Fibrillation in Blacks: The Atherosclerosis Risk in Communities (ARIC) Study

**DOI:** 10.1371/journal.pone.0110111

**Published:** 2014-10-16

**Authors:** Wobo Bekwelem, Jeffrey R. Misialek, Suma Konety, Scott D. Solomon, Elsayed Z. Soliman, Laura R. Loehr, Faye L. Lopez, Ervin R. Fox, Thomas H. Mosley, Alvaro Alonso

**Affiliations:** 1 Cardiovascular Division, University of Minnesota, Minneapolis, Minnesota, United States of America; 2 Division of Epidemiology and Community Health, University of Minnesota, Minneapolis, Minnesota, United States of America; 3 Department of Noninvasive Cardiology, Brigham and Women's Hospital, Boston, Massachusetts, United States of America; 4 Epicare, Wake Forest University School of Medicine, Winston-Salem, North Carolina, United States of America; 5 Department of Epidemiology, University of North Carolina at Chapel Hill, Chapel Hill, North Carolina, United States of America; 6 Division of Cardiology, University of Mississippi, Jackson, Mississippi, United States of America; 7 Division of Geriatrics, University of Mississippi, Jackson, Mississippi, United States of America; Case Western Reserve University, United States of America

## Abstract

**Background:**

Several studies have examined the link between atrial fibrillation (AF) and various echocardiographic measures of cardiac structure and function in whites and other racial groups but not in blacks. Exploring AF risk factors in blacks is important given that the lower incidence of AF in this racial group despite higher risk factors, is not completely explained.

**Methods:**

We examined the association of echocardiographic measures with AF incidence in 2283 blacks (64.5% women, mean age 58.8 years) free of diagnosed AF and enrolled in the Jackson cohort of Atherosclerosis Risk in Communities (ARIC) study, a prospective study of cardiovascular disease. Echocardiography was performed in 1993–1995, and incident AF was determined by electrocardiograms at a follow-up study exam, hospitalization discharge codes and death certificates through the end of 2009. Cox proportional hazards regression was used to estimate hazard ratios and 95% confidence intervals for AF associated with the echocardiographic measures, adjusting for age, sex, and known AF risk factors.

**Results:**

During an average follow-up of 13.5 years, 191 (8.4%) individuals developed AF. Left ventricular (LV) internal diameter 2-D (diastole) and percent fractional shortening of LV diameter displayed a U-shaped relationship with risk of AF, while left atrial diameter displayed a J-shaped nonlinear association. LV mass index was associated positively with AF. E/A ratio <0.7 or >1.5 and ejection fraction (EF <50%) were also associated with higher AF risk. These measures improved risk stratification for AF in addition to traditional risk factors, although not significantly {C-statistic of 0.767 (0.714–0.819) vs. 0.744 (0.691–0.797)}.

**Conclusions:**

In a community-based population of blacks, echocardiographic measures of cardiac structure and function are significantly associated with an increased risk of AF.

## Introduction

Atrial fibrillation (AF) is the most common cardiac arrhythmia in clinical practice, and its prevalence in the population is increasing [1]–[3]. Along with traditional risk factors, underlying structural and functional cardiac abnormalities such as chamber dilatation, diastolic dysfunction, and valvular problems are associated with higher AF risk [4]–[6]. Although blacks have a higher prevalence of AF risk factors than whites [7]–[8], they paradoxically have a lower risk for developing AF [2], [9]. While previous research has suggested that genetic factors might explain some of this racial disparity [10], the mechanisms by which these factors affect AF incidence still remain undetermined. Exploring AF risk factors in blacks is important given that the lower incidence of AF in this racial group despite higher risk factors is not completely explained. Understanding racial differences in risk factors for AF may help improve understanding of mechanisms and pathophysiology of the disease, prediction of AF in blacks, and prevention and treatment of AF in all populations.

Echocardiography is a useful modality in evaluating underlying structural and functional heart disease, and some studies have shown differences in echocardiographic parameters between whites and blacks [11]. The link between AF and various echocardiographic markers of cardiac structure and function in whites has been previously assessed [12]–[13]. To our knowledge, no study has examined this association in a large cohort of blacks with long term follow up for AF. Therefore, we examined standardized echocardiographic measures performed at the Jackson site of the Atherosclerosis Risk in Communities (ARIC) study.

We hypothesized that echocardiographic measures of cardiac structure and function, namely left atrial (LA) diameter, left ventricular (LV) internal diameter, LV mass index, depressed LV ejection fraction (LVEF), percent fractional shortening of 2D left ventricular diameter (%FS), and early-to-late (E/A) diastolic filling velocity ratio, are independently associated with incident AF in blacks.

## Methods

### Study Population

The ARIC Study has been previously described [14]. Briefly, the ARIC study is a population-based prospective study of cardiovascular disease (CVD) in a cohort of 15,792 individuals aged 45 to 64 years at enrollment (1987–1989) sampled from four US communities: Minneapolis, Minnesota; Washington County, Maryland; Forsyth County, North Carolina; and Jackson, Mississippi. Only blacks were recruited in Jackson. The baseline visit (visit 1) and three triennial visits - 1990–1992 (visit 2), 1993–1995 (visit 3), and 1996–1998 (visit 4), included interviews, laboratory measurements, and clinic examinations. Participants were also contacted annually by phone to obtain updated history. Echocardiograms were only performed at visit 3 (1993–1995) in the Jackson cohort (N = 2,622 black participants). Visit 3 was used as the baseline for these analyses. The ARIC study protocols were approved by the institutional review boards of each participating center, and informed consent was obtained from each study participant.

### Echocardiography

Details of the design and quality control measures for echocardiography have been described previously [15]. Echocardiographic parameters including 2D parasternal long axis LA diameter, LV internal diameter, LV posterior wall thickness (PWT), and interventricular septal thickness (IVST) were estimated at diastole. Relative wall thickness (RWT), defined as the ratio of the PWT multiplied by 2 and the LVID in diastole, was also calculated. The individual dimensions were used to estimate LV mass according to the American Society of Echocardiography (ASE) simplified cubed equation and were indexed by height^2^ to normalize heart size to body size [16]–[17]. This permitted categorization of individuals with increased LV mass index (>95 g/m^2^ in females and >115 g/m^2^ in males), as either concentric (RWT>0.42) or eccentric (RWT≤0.42) hypertrophy. Individuals with normal LV mass index were categorized as either concentric remodeling (RWT>0.42), or normal geometry (RWT≤0.42). Qualitative two-dimensional left ventricular systolic function (depressed {<50%} and normal) and Doppler mitral valve inflow consisting of early (E) and late (A) peak velocity were measured. Given that regional wall motion abnormalities were unlikely to be present in this population-derived cohort, %FS was used to better evaluate the relationship between LV function and AF. Doppler mitral inflow velocity time integral (VTI) of E wave and A wave were also measured. Intra- and intersonographer and intrareader correlation were excellent as assessed on a randomly selected 2% sample of participants (for example, 0.94, 0.88 and 0.98 respectively, for LV mass).

### Ascertainment of Incident Atrial Fibrillation

Only incident AF events occurring from visit 3 through December 31, 2009 were considered. Individuals with AF identified on or before visit 3 were excluded (N = 19). The methods used for AF ascertainment have previously been described in detail [9], [18]. AF was identified through hospital discharge codes (N = 182), electrocardiograms (ECG) performed at visit 4 (N = 2), death certificates (N = 1), and both hospital discharge codes and death certificates (N = 6). AF was identified when *International Classification of Diseases*, ninth revision, clinical modification (ICD-9-CM) code 427.31 or 427.32, or ICD-10 code I48, was listed. Previous analyses within this population have validated the diagnosis of incident AF based on hospital discharges [9].

Standard supine 12-lead resting ECGs were recorded after a 12-hour fast followed by a light snack and at least one hour after smoking tobacco or ingestion of caffeine. All ECGs automatically coded as AF were visually re-checked by a trained cardiologist to confirm the diagnosis. Left ventricular hypertrophy (LVH) based on Cornell voltage criteria [19], PR interval, and p wave indices [20] were also recorded.

### Measurement of other Covariates

At baseline (visit 3), weight, height, alcohol intake, cigarette smoking, blood pressure, and antihypertensive medication use were assessed using standardized methods [14]. Blood levels of calcium, magnesium, phosphorus, and uric acid were measured at visit 2. Definition of covariates are stated in the supplemental material (File S1).

### Statistical Analysis

**Figure 1:**
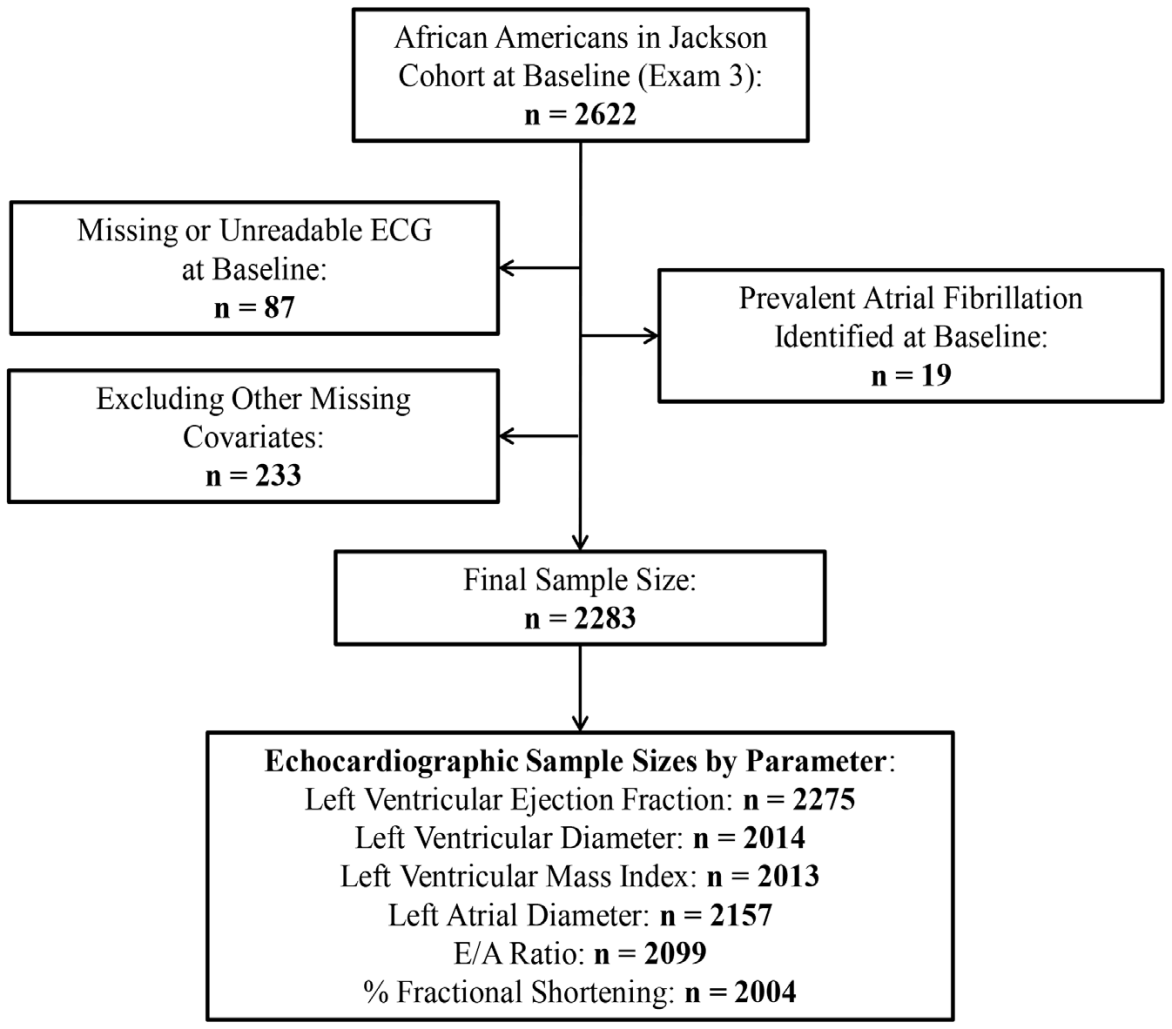
Inclusion and exclusion criteria, Atherosclerosis Risk in Communities Study Jackson Cohort, 1993–1995.

Of 2,622 blacks in the ARIC Jackson cohort at visit 3, we excluded 339 individuals (87 with missing or unreadable ECGs at baseline, 19 with prevalent AF, and 233 with missing covariates). The final sample included 2283 individuals. Due to technical limitations, different echocardiographic measurements were available for diverse sets of individuals, resulting in a variable sample size for each analysis (**figure 1**).

Cumulative incidence of AF by categories or quintiles of echocardiographic parameters were estimated using Kaplan-Meier curves. To estimate the association of echocardiographic measures with time to incident AF, we calculated hazard ratios (HRs) and 95% confidence intervals (CIs) using Cox proportional hazards models. Person-years at risk were calculated from visit 3 until date of development of AF, death, loss to follow up, or end of follow up (December, 2009), whichever occurred first. Initially, the shape of the association between each continuous echocardiographic measure and AF was explored using age- and sex-adjusted restricted cubic spline models (Figure S1 in File S1). In order not to assume linearity, LA diameter, LVID, %FS and LV mass index were modeled as quintiles. Individuals were dichotomized into two groups for LVEF: depressed (<50%) or normal. Mitral early-to-late (E/A) diastolic filling velocity ratio was divided into 3 categories (<0.7, 0.7–1.5, and >1.5), with 0.7–1.5 used as the reference point for all analyses. In an initial model, we adjusted for age and sex. A second model additionally adjusted for the recently described CHARGE-AF risk score [21], which consists of known AF risk factors including age, height, weight, current smoking, systolic blood pressure, diastolic blood pressure, anti-hypertensive medications, diabetes, prevalent HF, and prevalent MI; and estimates cumulative risk of AF over 5 years. The proportional hazards assumption was tested using time interaction terms and inspection of log-negative log survival curves. The assumption was satisfied for all parameters except the E/A ratio. To address this issue, follow-up for the E/A ratio analysis was split at the midpoint into two time periods (0–8 years and >8 years to the end of follow-up) at the midpoint of average follow up time, and HRs were calculated for each period. Interactions by sex and HTN (a major risk factor for AF) with these echocardiographic parameters were examined. Interaction between LVH (by voltage criteria on ECG) and LV diameter; and between LV mass index and RWT was also examined.

Furthermore, we determined whether echocardiographic measures (modeled as continuous variables) improve the predictive ability of the CHARGE AF risk score measuring improvement in C-statistic for survival analysis over ten years [22]. To determine whether individuals were reclassified correctly rather than due to chance, we calculated the Net Reclassification Improvement (NRI), using <5.0%, 5.0%–10.0%, >10.0% risk as cutoff points over ten years [23]. To avoid associations resulting from reverse causation (AF leading to changes in echocardiographic parameters) and to avoid including possible undetected prevalent AF cases as new incident cases, we conducted a sensitivity analysis excluding cases of AF occurring in the first 2 years after echocardiographic measurements.

Finally, in secondary analyses, we added ECG measures of increased LA size (defined as P wave duration ≥120 ms) and ECG-LVH (Cornell voltage criteria) to models with echocardiography measurements to determine whether ECG measures added predictive value. The risk of AF based on the presence of concentric hypertrophy, eccentric hypertrophy, or concentric remodeling compared to normal geometry was determined. We further adjusted covariate analyses models for PR interval, calcium, magnesium, phosphorus, uric acid and alcohol use. Analyses were performed using SAS, version 9.2; SAS Institute Inc. All p values were 2-sided.

## Results

**Table 1** shows selected clinical, echocardiographic and electrocardiographic measures of ARIC Jackson cohort participants at visit 3 by incident AF status. In this population, individuals who developed AF were more likely to be older males, to have higher CHARGE-AF risk scores, larger LA, heavier LV, and more systolic or diastolic dysfunction. They were also more likely to have LVH based on Cornell voltage criteria and LA enlargement by P wave indices.

**Table 1:** Baseline characteristics with incident atrial fibrillation (AF) events through 2009 follow-up, Atherosclerosis Risk in Communities Study Jackson Cohort, 1993–1995.

		No AF during follow-up	Incident AF	
		(n = 2092)	(n = 191)	P-Value
Clinical Measures*				
**Age, years**		59±5.6	61±5.7	<0.0001
**Sex, male**		729 (34.9)	82 (42.9)	0.03
**Body Mass Index, kg/m^2^**		30.4±6.3	32.1±6.8	0.0004
**Current Smoking**		399 (19.1)	54 (28.3)	0.002
**High School Graduate**		578 (27.6)	61 (31.9)	<0.0001
**High-Density Cholesterol, mg/dL**		57±18.5	50±15.6	<0.0001
**Systolic Blood Pressure, mmHg**		130±19.9	136±22.2	<0.0001
**Diastolic Blood Pressure, mmHg**		77±10.4	76±12.4	0.17
**Anti-Hypertensive Medications**		1041 (49.8)	138 (72.3)	<0.0001
**Digoxin**		30 (1.4)	11 (5.8)	<0.0001
**Prevalent Diabetes**		462 (22.1)	79 (41.4)	<0.0001
**Prevalent Heart Failure**		124 (5.9)	27 (14.1)	<0.0001
**Prevalent Coronary Heart Disease**		84 (4.0)	19 (10.0)	0.0002
**Current alcohol use**		622 (29.7)	63 (33)	0.15
**CHARGE-AF Risk Score, % (5 years)**		1.5±1.6	2.9±2.4	<0.0001
**Serum Calcium, mg/dL**		9.5 (0.5)	9.5 (0.4)	0.92
**Serum Magnesium, mEq/L**		1.6 (0.2)	1.6 (0.2)	0.82
**Serum Phosphorus, mmol/L**		4.2 (0.4)	4.1 (0.5)	0.04
**Uric Acid, mg/dL**		6.6 (1.7)	7.2 (2.0)	<0.0001
Echocardiographic Measures*				
**Left Atrial Diameter, cm**		3.3±0.5	3.6±0.6	<0.0001
**Left Ventricular Diameter (Diastole), cm**		4.4±0.6	4.4±0.8	0.07
**Left Ventricular Mass Index, g/m^2^**		84.4±24.8	99.4±38.2	<0.0001
**Depressed Left Ventricular Ejection Fraction (EF <50%)**		39 (1.9)	13 (6.8)	<0.0001
**Mitral Early-to-Late (E/A) Diastolic Filling Velocity Ratio**				<0.0001
	<0.7	154 (7.4)	28 (14.7)	
	>1.5	125 (6.0)	17 (8.9)	
**% Fractional Shortening of Left Ventricular Diameter**		34.6±8.9	33.4±10.5	0.10
**Relative Wall Thickness**		0.56 (0.13)	0.61 (0.16)	<0.0001
**Electrocardiographic Measures** *				
**Left Atrial Enlargement, P Wave Index**		569 (27.8)	69 (37.1)	0.01
**Left Ventricular Hypertrophy, Cornell Voltage Criteria**		119 (5.8)	23 (12.3)	0.001
**PR Interval, msec**		172 (28)	171 (40)	0.69

* Data given as mean±SD or n (%).

**Figure 2:**
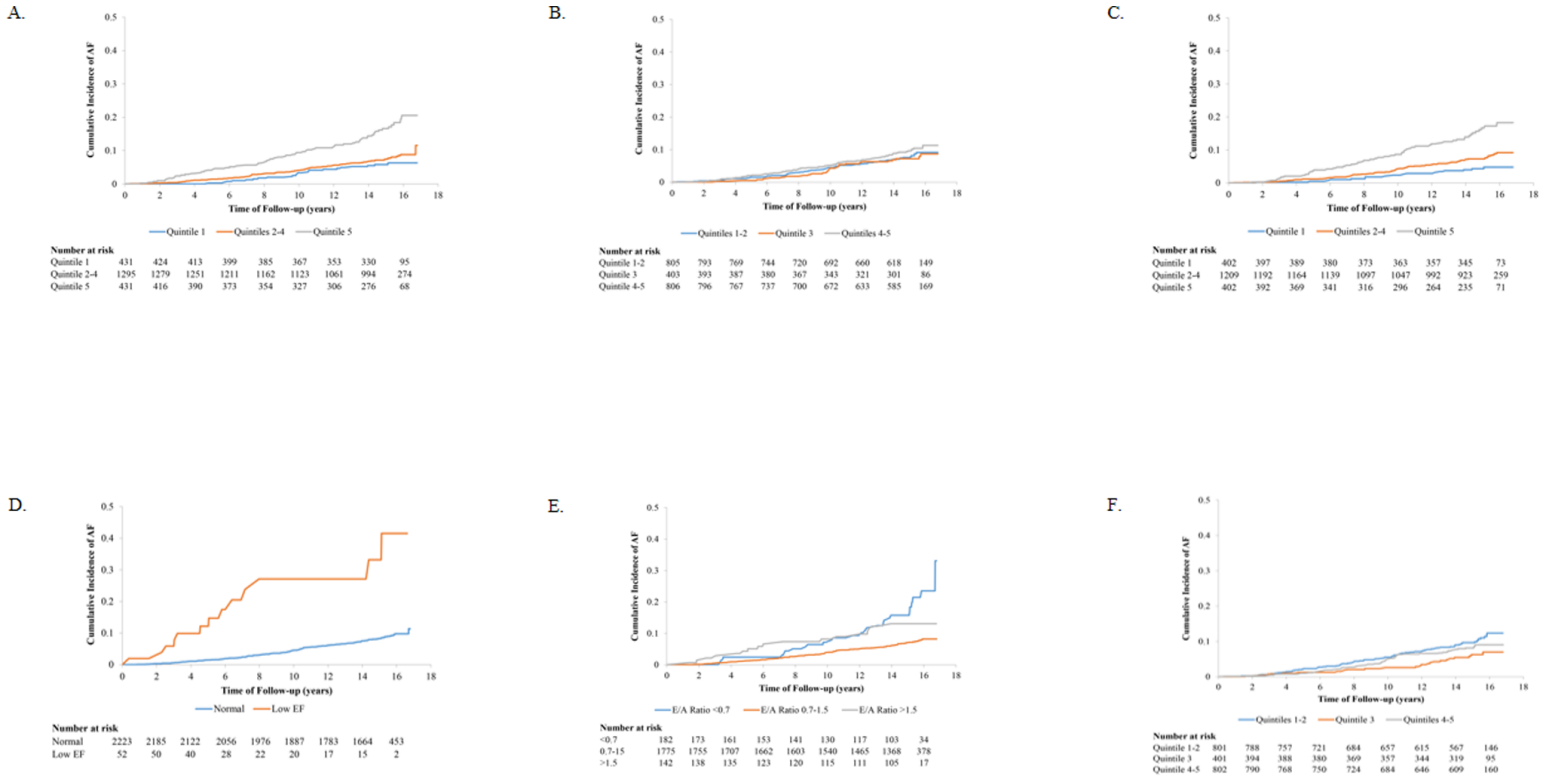
Kaplan-Meier curves for selected echocardiographic parameters, Atherosclerosis Risk in Communities Study Jackson Cohort, 1993–2009. A: Left Atrial Diameter. B: Left Ventricular Diameter (Diastole). C: Left Ventricular Mass Index (g/m^2^). D: Left Ventricular Ejection Fraction. E: E/A Ratio. F: % Fractional Shortening of the Left Ventricular Diameter.

During a total 30,826 person-years of follow-up (mean follow-up 13.5 years), we identified 191 incident cases of AF. After adjustment for age and sex, longer LA diameter was associated with an increased risk of AF in a J-shaped pattern (Figure S1 in File S1). Compared to those in the 1^st^ quintile, the HR (95% CI) of AF for those in the 5^th^ quintile was 2.46 (1.51–4.02), after adjustment for the CHARGE-AF score. Similarly, LV mass index was linearly associated with AF risk: HR (95% CI) comparing the 5^th^ to the 1^st^ quintile was 2.81 (1.61–4.91) after adjustment for the CHARGE-AF score (**Table 2**). There was no significant interaction between LV mass index and RWT (p = 0.54). On the other hand, LV diameter showed a U-shaped relationship with incident AF (Figure S1 in File S1), with increased risk among individuals with small and those with large diameter. Risk was lowest at 2^nd^ quintile (HR 0.49, 95% CI 0.29–0.83 compared to the 1^st^ quintile) (**Table 2** **, Model 2**). %FS also showed a U-shaped relationship with incident AF (Figure S1 in File S1), with risk lowest among individuals in the third quintile (32.41–36.55) of %FS. Reduced LVEF was associated with increased risk of AF (HR 3.46, 95% CI 1.88–6.36; **Table 2** **, Model 2**). Finally, diastolic dysfunction based on an E/A ratio <0.7 and >1.5 was also associated with increased AF risk compared to an E/A ratio 0.7–1.5, though the magnitude of the association differed over the entire follow-up (**Table 2**). Additionally adjusting for educational level, HDL cholesterol and digoxin use and incident HF and MI as time-dependent covariates did not change the results (Table S1 in File S1). Analyzing hazard ratios for AF by sex-specific quintiles did not significantly change the results (Table S2 in File S1). Kaplan-Meier curves showing cumulative incidence of AF by quintiles and categories of echocardiographic parameters are shown in **figure 2**. Due to a small number of AF cases, quintiles were divided into 3 groups in the Kaplan-Meier curves.

**Table 2:** Hazard ratios (HR) and 95% confidence intervals (CI) for the association of echocardiographic parameters in quintiles with time to incident atrial fibrillation (AF), ARIC Jackson Cohort, 1993–2009.

Parameter	AF Cases | N	Group/Quintile	Model 1† HR (95% CI)	Model 2‡ HR (95% CI)
Left Atrial Diameter, cm	23 | 431	1.54–2.93	Reference	Reference
	28 | 432	>2.93–3.21	1.21 (0.70–2.10)	1.14 (0.66–1.98)
	26 | 432	>3.21–3.46	1.11 (0.63–1.94)	1.05 (0.60–1.85)
	40 | 431	>3.46–3.78	1.76 (1.05–2.94)	1.62 (0.97–2.71)
	65 | 431	>3.78–5.18	3.06 (1.90–4.93)	2.46 (1.51–4.02)
**P for Trend** *			<0.0001	<0.0001
Left Ventricular Mass Index, g/m^2^	17 | 402	27.91–65.03	Reference	Reference
	26 | 403	65.04–75.77	1.42 (0.77–2.61)	1.40 (0.76–2.58)
	30 | 403	75.78–86.96	1.70 (0.94–3.08)	1.66 (0.91–3.00)
	33 | 403	86.97–102.86	1.87 (1.04–3.36)	1.70 (0.95–3.06)
	55 | 402	102.87–316.60	3.52 (2.04–6.08)	2.81 (1.61–4.91)
**P for Trend** *			<0.0001	0.0001
Left Ventricular Diameter (Diastole), cm	39 | 402	2.30–3.87	Reference	Reference
	21 | 403	>3.87–4.21	0.50 (0.29–0.85)	0.49 (0.29–0.83)
	28 | 403	>4.21–4.48	0.72 (0.44–1.17)	0.68 (0.42–1.11)
	34 | 403	>4.48–4.81	0.80 (0.50–1.28)	0.72 (0.45–1.15)
	39 | 403	>4.81–7.19	1.03 (0.65–1.62)	0.78 (0.48–1.24)
**P for Trend** *			0.42	0.72
% Fractional Shortening of Left Ventricular Diameter	44 | 400	1.51–27.46	Reference	Reference
	32 | 401	27.47–32.40	0.69 (0.44–1.08)	0.79 (0.50–1.25)
	23 | 401	32.41–36.55	0.45 (0.27–0.75)	0.50 (0.30–0.83)
	29 | 401	36.56–41.75	0.62 (0.38–0.99)	0.71 (0.44–1.15)
	33 | 401	41.76–78.21	0.65 (0.41–1.02)	0.72 (0.45–1.15)
**P for Trend** *			0.06	0.14
Left Ventricular Ejection Fraction (LVEF)	175 | 2223	*Normal (≥50%)*	Reference	Reference
	13 | 52	*Low LVEF (<50%)*	5.16 (2.91–9.14)	3.46 (1.88–6.36)
Mitral Early-to-Late (E/A) Diastolic Filling Velocity Ratio: 0–8 years	8 | 182	<0.7	1.37 (0.64–2.93)	1.09 (0.50–2.37)
	45 | 1775	0.7–1.5	Reference	Reference
	10 | 142	>1.5	3.56 (1.78–7.14)	3.59 (1.79–7.20)
>8 years to the end of follow-up	20 | 141	<0.7	2.81 (1.69–4.67)	2.47 (1.47–4.15)
	73 | 1603	0.7–1.5	Reference	Reference
	7 | 120	>1.5	1.51 (0.69–3.31)	1.51 (0.69–3.30)

* Linear trend in quintile number.

† Model 1 - adjusted for age and sex.

‡ Model 2 – adjusted for Model 1 + CHARGE risk score.

After adjustment for the CHARGE-AF risk score, LA enlargement by P wave index on ECG was associated with a 35% increased risk for incident AF {HR (95% CI) = 1.35 (1.00–1.82)}. Similarly, LVH using Cornell voltage criteria was also associated with increased AF risk {HR (95% CI) = 2.28 (1.47–3.53)} (Tables S3 and S4 in File S1). For all models examined, there was no significant evidence of interaction by HTN or sex. There was no significant interaction between LVH (Cornell voltage criteria) and LV diameter (P = 0.48). In a sensitivity analysis, the above models were further adjusted for phosphorus, magnesium, uric acid, calcium, alcohol use and PR interval on ECG both individually and all in the same model. The results were unchanged from those from the above models (data not shown).

Addition of individual echocardiographic variables to the CHARGE AF score resulted in a modest, non-statistically significant improvement in the C-statistic for prediction of AF over a 10-year follow-up (**Table 3**). NRI for adding LA diameter to the CHARGE-AF risk score was 0.12 (p = 0.01); other echocardiographic variables did not improve reclassification over ten years (**Table 3**). Addition of ECG variables to models containing similar echocardiographic variables did not significantly improve C statistic or NRI (Tables S5 and S6 in File S1).

**Table 3:** C-Statistic and net reclassification index (NRI) for the association of echocardiographic parameters (as continuous variables) with time to incident atrial fibrillation, ARIC Jackson Cohort, 1993–2009.

ECHO variable	C-statistic (95% CI) without ECHO variable	C-statistic (95% CI) with ECHO variable	NRI (0.05,0.10)	P-value
Left Atrial Diameter (cm)	0.718 (0.671–0.766)	0.741 (0.691–0.791)	0.12	0.01
Left Ventricular Diameter (Diastole) (cm)	0.740 (0.693–0.788)	0.750 (0.700–0.799)	0.03	0.41
Left Ventricular Mass Index (g/m^2^)	0.740 (0.693–0.787)	0.752 (0.706–0.799)	0.04	0.31
Depressed Left Ventricular Ejection Fraction (LVEF <50%)	0.726 (0.681–0.772)	0.739 (0.694–0.785)	0.03	0.36
Mitral Early-to-Late (E/A) Diastolic Filling Velocity Ratio	0.733 (0.683–0.783)	0.735 (0.685–0.785)	0.003	0.90
% Fractional Shortening of Left Ventricular Diameter	0.740 (0.692–0.787)	0.750 (0.705–0.795)	0.03	0.44
All Echo variables: n = 1773; AF cases = 72	0.744 (0.691–0.797)	0.767 (0.714–0.819)		

When we examined all the echocardiographic variables in a single model, C-statistic improved from 0.744 (0.691–0.797) to 0.767 (0.714–0.819). Addition of both echocardiographic and ECG variables in the same model produced a similar result: C-statistic 0.768 (0.715–0.821). Sample size for these analyses were smaller (1773 individuals with 72 AF cases) as these were the only individuals who had all the echocardiographic and ECG measures available. In a sensitivity analysis excluding AF cases identified during the first 2 years of follow-up, results were unchanged (data not shown).

Finally, we examined four RWT-LV mass index groups to assess their relationship to AF. After adjustment for age, sex and the CHARGE-AF risk score, concentric and eccentric hypertrophy were associated with increased AF risk, compared with normal geometry, HR 3.17 (1.35–7.48) and 4.87 (1.37–17.35) respectively. There was no increased risk of AF with concentric remodeling, HR 1.72 (0.75–3.93).

## Discussion

This population-based prospective study of blacks aged 51–70 years, found that echocardiographic measures of cardiac structure and function are associated with an increased risk of AF, independent of sex, lifestyles, clinical measures and chronic diseases. We found that the association of some echocardiographic measures (LA diameter, LV diameter, and %FS) with AF risk is complex and non-linear. Specifically, LA diameter showed a J-shaped association, with AF risk markedly increasing in the top quintile (>3.78–5.18 cm). Several studies have shown that LA diameter is a risk factor for AF [11], [24], [25]. It has also been shown that LA diameter is smaller in blacks than whites, and that this difference could contribute to lower AF risk in blacks [11], [26]. Marcus et al. [11] found that mean LA diameter was 4.26 cm in blacks vs. 4.33 cm in whites (p = 0.0026). Two prior reports using data from the Framingham and Cardiovascular Health studies have demonstrated a link between LA diameter and incident AF mostly in whites [24]–[25]; however, non-linear associations were not evaluated in these reports. Rosenberg et al. [12] evaluated non-linear associations between diastolic parameters and AF also in whites, but found only a linear association between LA diameter and AF risk. Only one prior study has compared echocardiographic parameters and risk of AF in blacks and whites [11], however they only included 476 blacks with 6 AF cases. Since our study is the first to report this J-shaped relationship between LA diameter and AF risk, it remains to be seen if this finding is particular to blacks.

In a similar way, in the present study, LV diameter and %FS both showed a U-shaped relationship with incident AF, with risk of AF lowest at the midpoint of LV diameter and %FS 32.41–36.55. The increased risk with smaller LV diameter may be explained by the effect of hypertensive heart disease leading to a thicker left ventricle and hence smaller internal diameter. In our analyses, there was however no effect modification of LVH (Cornell voltage criteria) on LV diameter. Both concentric hypertrophy (which can be caused by pressure overload from hypertension), and eccentric hypertrophy (caused by volume overload) were associated with increased AF risk. %FS is a surrogate of cardiac contractility and although it is dependent on preload, it is a long established method of estimating LV function [37]. It only fails in individuals with regional wall motion abnormalities which is not expected to be present in this population-derived cohort. That there was a lower risk of AF in patients with %FS 32.41–36.55, compared to those with lower %FS is not surprising, as low LVEF (another marker of reduction in myocardial function) was associated with lower AF risk. Increased catecholamine release is associated with AF [38]. In high catecholamine states, there may be hyperdynamic cardiac function, and hence %FS may be increased. This might explain the link between increased %FS and AF; hence the U shaped relationship between %FS and AF. Also, LV mass index (a marker of LVH), diastolic dysfunction measured by E/A ratio <0.7 and >1.5, and reduced LV systolic function were associated with increased risk of AF [12], [27], [28]. These measures (except LV diameter), both individually and together in a single model improve risk stratification and discriminative ability over traditional risk factors for AF in blacks, although this improvement was not statistically significant. Addition of ECG measures did not significantly add to risk stratification. Although these measures are associated with risk of AF in blacks, the associations between these measures and AF in blacks are similar to those in whites [11], [12], [24], [25]; hence these measures alone are unlikely to explain the racial differences found in AF.

The complex mechanisms underlying development of AF remain incompletely understood. AF is thought to result from presence of multiple wave fronts conducted to the ventricles from the atria and requires triggers [29]. The regular impulses produced by the sinus node to provide rhythmic contraction of the heart are overwhelmed by these rapid randomly generated multiple wave fronts [30]–[31]. There have been a few mechanisms suggested by which echocardiographic markers of cardiac structure and function may increase risk of developing AF. Increased LA size has been thought to increase AF risk as a result of stretch of the atrial appendage which leads to remodelling of the anatomy and physiology of the left atrium and increases dispersion of atrial refractoriness [25], [32], [33]. Impairment of LV end-diastolic filling pressure and LA emptying caused by LV size, systolic and diastolic dysfunction can also lead to development of AF by worsening atrial structural remodeling. AF itself reduces atrial contractility, contributing to atrial dilatation and more remodeling, thus contributing to the progressive and perpetuating nature of this arrhythmia [34]. HTN, CHD, age, inflammation and other risk factors act as triggers and propagating factors directly influencing these measures of cardiac structure and function increasing AF risk. For example, age- and inflammation-related fibrosis can cause increased atrial size, and HTN and CHD lead to increased LV mass and cardiomyopathy. However, a positive association of echocardiographic measures with AF persisted even after we accounted for these traditional risk factors.

Our study has some limitations. First, we did not have volumetric measures of atrial size. The limitations of assessment of atrial size in M-mode or 2-D are widely recognized, and it is accepted that volumetric measures are better. Second, markers of HF (NT-proBNP), thyroid dysfunction (TSH) and renal failure (eGFR), which are recognized risk factors for AF, were not available. Hence, we could not adjust for them. However, we do not expect that adjusting for these factors would have changed our overall findings, since accounting for HF and other traditional risk factors for AF did not completely explain the associations we found. Third, most of our AF cases are identified due to hospitalizations, which may not account for AF managed in outpatient settings. Also, as AF is often transient, asymptomatic patients may have been missed. Therefore, there is likely an under ascertainment of incident AF cases. However, AF cases through hospitalizations in ARIC have been validated [9], [24]. Fourth, since echocardiograms were performed between 1993 and 1995, measures needed to properly classify diastolic function by current ASE guidelines were not available. Prior studies, however, have shown that E/A ratio of <0.7 and >1.5 compared to 0.7-1.5 is a marker for diastolic dysfunction and risk factor for CVD [35]–[36]. Also, while individuals with E/A ratio>1.5 could potentially have normal filling, most in this category have demonstrated increased CVD risk [35]–[36]. Since not all echocardiographic measures were available for all individuals in the analysis, we were unable to examine the overall relationship with AF and all measures in a single model. Lastly, whites did not have echocardiograms performed at visit 3, so we do not have direct racial comparisons. Despite its limitations, this study is novel and has important strengths, including the relatively large sample size, extended follow-up, and availability of information on potential confounders.

### Conclusions

We have shown that echocardiographic measures of cardiac structure and function in blacks are associated with an increased risk of AF independent of traditional risk factors. Although these measures individually and in a single model modestly improve risk stratification and discriminative ability of AF in this population, the only statistically significant improvement was for LA diameter. Addition of ECG measures did not significantly add to risk stratification. Further research is needed to determine if blacks at increased risk based on echocardiographic measures can be targeted with therapies to attempt to reduce incidence of AF. Lastly, large prospective studies with direct racial comparisons of associations of imaging modalities with AF are warranted.

## Supporting Information

File S1 **Supporting information.** Tables, Figures, and description of terms in methods section of paper.
(DOCX)
