# Supplementary material for: Echocardiographic Measures of Cardiac Structure and Function Are Associated with Risk of Atrial Fibrillation in Blacks: The Atherosclerosis Risk in Communities (ARIC) Study
Source: PLoS One. 2014 Oct 16;9(10):e110111. doi: 10.1371/journal.pone.0110111 (PMC4199625; doi:10.1371/journal.pone.0110111)
Supplement: File S1 — Supporting information. Tables, Figures, and description of terms in methods section of paper. (DOCX) [file pone.0110111.s001.docx]

**SUPPORTING INFORMATION FILE S1**

**Echocardiographic measures of cardiac structure and function are associated with risk of atrial fibrillation in Blacks: The Atherosclerosis Risk in Communities (ARIC) Study**

Wobo Bekwelem, Jeffrey R. Misialek, Suma Konety, Scott D. Solomon, Elsayed Z. Soliman, Laura R. Loehr, Faye L. Lopez, Ervin R. Fox, Thomas H. Mosley, Alvaro Alonso

**Expanded Methods:**

**Definition of** **Covariates:**

HTN was defined as a blood pressure of >140/90 mm Hg or current use of antihypertensive medication.

Diabetes was defined as a fasting serum glucose level >126 mg/dL, nonfasting glucose level >200 mg/dL, report of a physician diagnosis of diabetes or current use of diabetes medication.

Prevalent coronary heart disease (CHD) was defined by electrocardiographic evidence of previous myocardial infarction (MI), history of physician diagnosed MI, or previous coronary revascularization procedure (bypass, angioplasty).

Prevalent HF was defined by the reported current intake of HF medication at visit 3 or evidence of manifest HF with presence of specific cardiac and pulmonary symptoms.

**Table S1.** Hazard ratios (HR) and 95% confidence intervals (CI) for atrial fibrillation (AF) by quintiles of echocardiographic parameters, ARIC Jackson Cohort, 1993-1995

| **Parameter** | **AF Cases \| N** | **Group/Quintile** | **Model 3 HR (95% CI)** | **Model 4 HR (95% CI)** |
| --- | --- | --- | --- | --- |
| Left Atrial Diameter, cm | 23 \| 431 | 1.54-2.93 | Reference | Reference |
|  | 28 \| 432 | >2.93-3.21 | 1.11 (0.64-1.94) | 1.25 (0.72-2.18) |
|  | 26 \| 432 | >3.21-3.46 | 0.99 (0.56-1.73) | 0.98 (0.56-1.72) |
|  | 40 \| 431 | >3.46-3.78 | 1.46 (0.87-2.45) | 1.32 (0.78-2.22) |
|  | 65 \| 431 | >3.78-5.18 | 2.18 (1.33-3.58) | 1.89 (1.15-3.09) |
| **P for Trend*** | | | 0.0003 | 0.01 |
| Left Ventricular Mass Index, g/m^2^ | 17 \| 402 | 27.91-65.03 | Reference | Reference |
|  | 26 \| 403 | 65.04-75.77 | 1.30 (0.71-2.41) | 1.09 (0.59-2.02) |
|  | 30 \| 403 | 75.78-86.96 | 1.48 (0.81-2.69) | 1.29 (0.71-2.36) |
|  | 33 \| 403 | 86.97-102.86 | 1.57 (0.87-2.82) | 0.98 (0.54-1.80) |
|  | 55 \| 402 | 102.87-316.60 | 2.39 (1.36-4.20) | 1.27 (0.71-2.26) |
| **P for Trend*** | | | 0.001 | 0.54 |
| Left Ventricular Diameter (Diastole), cm | 39 \| 402 | 2.30-3.87 | Reference | Reference |
|  | 21 \| 403 | >3.87-4.21 | 0.50 (0.29-0.85) | 0.45 (0.26-0.77) |
|  | 28 \| 403 | >4.21-4.48 | 0.65 (0.40-1.07) | 0.54 (0.33-0.90) |
|  | 34 \| 403 | >4.48-4.81 | 0.69 (0.43-1.11) | 0.63 (0.39-1.01) |
|  | 39 \| 403 | >4.81-7.19 | 0.74 (0.46-1.19) | 0.49 (0.30-0.80) |
| **P for Trend*** | | | 0.51 | 0.05 |
| % Fractional Shortening of Left Ventricular Diameter | 44 \| 400 | 1.51-27.46 | Reference | Reference |
|  | 32 \| 401 | 27.47-32.40 | 0.82 (0.52-1.31) | 1.41 (0.87-2.27) |
|  | 23 \| 401 | 32.41-36.55 | 0.52 (0.31-0.86) | 0.90 (0.53-1.51) |
|  | 29 \| 401 | 36.56-41.75 | 0.75 (0.46-1.22) | 1.22 (0.75-2.00) |
|  | 33 \| 401 | 41.76-78.21 | 0.77 (0.48-1.23) | 1.38 (0.85-2.24) |
| **P for Trend*** | | | 0.23 | 0.34 |
| Left Ventricular Ejection Fraction (LVEF) | 175 \| 2223 | *Normal (≥50%)* | Reference | Reference |
|  | 13 \| 52 | *Low LVEF (<50%)* | 2.97 (1.60-5.51) | 1.37 (0.74-2.54) |
| Mitral Early-to-Late (E/A) Diastolic Filling Velocity Ratio:  0-8 years | 8 \| 182 | <0.7 | 0.88 (0.41-1.93) | 0.77 (0.35-1.71) |
|  | 45 \| 1775 | 0.7-1.5 | Reference | Reference |
|  | 10 \| 142 | >1.5 | 3.67 (1.83-7.38) | 3.78 (1.87-7.64) |
| >8 years to the end of follow-up | 20 \| 141 | <0.7 | 2.14 (1.26-3.63) | 1.44 (0.84-2.48) |
|  | 73 \| 1603 | 0.7-1.5 | Reference | Reference |
|  | 7 \| 120 | >1.5 | 1.60 (0.73-3.50) | 2.27 (1.02-5.05) |

***** Linear trend in quintile number.

† Model 3 – adjusted for Model 2 + Education Level, HDL Cholesterol, & Digoxin

‡ Model 4 – adjusted for Model 3 + incident HF and MI as time-dependent covariates

**Table S2.** Hazard ratios (HR) and 95% confidence intervals (CI) for atrial fibrillation (AF) by sex-specific quintiles of echocardiographic parameters, ARIC Jackson Cohort, 1993-1995

| **Parameter** | **AF Cases \| N** | **Model 1 HR (95% CI)** | | **Model 2 HR (95% CI)** | |  |
| --- | --- | --- | --- | --- | --- | --- |
| Left Atrial Diameter, cm | 23 \| 431 | Reference | | Reference | |  |
|  | 27 \| 432 | 1.18 (0.68-2.06) | | 1.13 (0.65-1.97) | |  |
|  | 28 \| 431 | 1.19 (0.69-2.07) | | 1.13 (0.65-1.96) | |  |
|  | 40 \| 432 | 1.78 (1.07-2.97) | | 1.65 (0.99-2.76) | |  |
|  | 64 \| 431 | 3.10 (1.93-5.00) | | 2.49 (1.53-4.07) | |  |
| **P for Trend*** | | | <0.0001 | | <0.0001 | |
| Left Ventricular Mass Index, g/m^2^ | 16 \| 401 | Reference | | Reference | |  |
|  | 26 \| 403 | 1.61 (0.86-3.00) | | 1.58 (0.85-2.95) | |  |
|  | 32 \| 403 | 1.96 (1.08-3.57) | | 1.90 (1.04-3.45) | |  |
|  | 30 \| 403 | 1.85 (1.01-3.39) | | 1.69 (0.92-3.11) | |  |
|  | 57 \| 403 | 4.07 (2.33-7.08) | | 3.24 (1.84-5.71) | |  |
| **P for Trend*** | | | <0.0001 | | <0.0001 | |
| Left Ventricular Diameter (Diastole), cm | 36 \| 402 | Reference | | Reference | |  |
|  | 22 \| 403 | 0.62 (0.36-1.05) | | 0.58 (0.34-0.98) | |  |
|  | 36 \| 403 | 1.03 (0.65-1.64) | | 0.96 (0.60-1.52) | |  |
|  | 28 \| 403 | 0.80 (0.49-1.31) | | 0.73 (0.44-1.19) | |  |
|  | 39 \| 403 | 1.29 (0.82-2.03) | | 0.99 (0.62-1.58) | |  |
| **P for Trend*** | | | 0.20 | | 0.78 | |
| % Fractional Shortening of Left Ventricular Diameter | 41 \| 400 | | Reference | | Reference | |
|  | 29 \| 402 | | 0.61 (0.38-0.98) | | 0.73 (0.45-1.18) | |
|  | 28 \| 400 | | 0.59 (0.36-0.95) | | 0.68 (0.42-1.10) | |
|  | 30 \| 402 | | 0.59 (0.37-0.95) | | 0.68 (0.42-1.10) | |
|  | 33 \| 400 | | 0.63 (0.40-1.00) | | 0.72 (0.45-1.15) | |
| **P for Trend*** | | | 0.08 | | 0.17 | |

**Table S3.** Hazard ratios (HR) and 95% confidence intervals (CI) for the association of electrocardiographic parameters with time to incident atrial fibrillation (AF), ARIC Jackson Cohort, 1993-2009

| **Parameter** | **AF Cases \| N** | **Model 1 HR (95% CI)** | **Model 2 HR (95% CI)** |
| --- | --- | --- | --- |
| Left Atrial Enlargement,  P-Wave Index | 69 \| 638 | 1.44 (1.07-1.95) | 1.35 (1.00-1.82) |
| Left Ventricular Hypertrophy, Voltage Criteria | 23 \| 142 | 2.27 (1.47-3.52) | 2.28 (1.47-3.53) |

***** Model 1 - adjusted for age and sex

† Model 2 – adjusted for Model 1 + CHARGE risk score

**Table S4.** Hazard ratios (HR) and 95% confidence intervals (CI) for atrial fibrillation (AF) by electrocardiographic parameters, ARIC Jackson Cohort, 1993-1995

| **Parameter** | **AF Cases \| N** | **Model 3 HR (95% CI)** | **Model 4 HR (95% CI)** |
| --- | --- | --- | --- |
| Left Atrial Enlargement,  P-Wave Index | 69 \| 638 | 1.30 (0.96-1.75) | 1.36 (1.01-1.84) |
| Left Ventricular Hypertrophy, Voltage Criteria | 23 \| 142 | 2.11 (1.35-3.30) | 1.02 (0.64-1.65) |

***** Model 3 – adjusted for Model 2 + Education Level, HDL Cholesterol, & Digoxin

† Model 4 – adjusted for Model 3 + incident HF and MI as time-dependent covariates

**Table S5.** C-Statistic and net reclassification index (NRI) for the association of electrocardiographic (ECG) parameters with time to incident atrial fibrillation, ARIC Jackson Cohort, 1993-2009

| **ECG variable** | **C-statistic (95 % CI) without ECG variable** | **C-statistic (95 % CI) with ECG variable** | **NRI (0.05,0.10)** | **P-value** |
| --- | --- | --- | --- | --- |
| **LA enlargement** | 0.715  (0.667-0.763) | 0.716  (0.667-0.765) | 0.02 | 0.42 |
| **LV Hypertrophy** | 0.744  (0.696-0.791) | 0.755  (0.708-0.801) | 0.03 | 0.36 |

**Table S6.** C-Statistic and net reclassification index (NRI) for the association of electrocardiographic (ECG) and echocardiographic parameters with time to incident atrial fibrillation, ARIC Jackson Cohort, 1993-2009

| **ECG variable** | **C-statistic (95 % CI) with ECG + Echo variable** | **NRI (0.05,0.10)** | **P-value** |
| --- | --- | --- | --- |
| **LA enlargement** | 0.739 (0.688-0.790) | 0.10 | 0.04 |
| **LV Hypertrophy** | 0.760  (0.715-0.806) | 0.08 | 0.10 |

*For these results, LA enlargement included the LA diameter as a quadratic while LV hypertrophy included LV mass index as a linear variable.

**Figure S1. Association between atrial fibrillation (AF) risk with (a) left atrial diameter, (b) left ventricular mass index, (c) left ventricular diameter (diastole), and (d) % fractional shortening of the left ventricular diameter using restricted cubic spline (RCS) plot with 5 knots, adjusted for age and sex.**

**a.)**

**b.)**

**c.)**

**d.)**
